# Priority of Fibular Reconstruction in Patients with Oral Cavity Cancer Undergoing Segmental Mandibulectomy

**DOI:** 10.1371/journal.pone.0094315

**Published:** 2014-04-10

**Authors:** Chih-Hung Lin, Chung-Jan Kang, Chung-Kan Tsao, Christopher Glenn Wallace, Li-Yu Lee, Chien-Yu Lin, Hung-Ming Wang, Shu-Hang Ng, Tzu-Chen Yen, Chun-Ta Liao

**Affiliations:** 1 Department of Plastic and Reconstructive Surgery, Chang Gung Memorial Hospital and Chang Gung University, Taoyuan, Taiwan, R.O.C.; 2 Department of Head and Neck Oncology Group, Chang Gung Memorial Hospital and Chang Gung University, Taoyuan, Taiwan, R.O.C.; 3 Department of Otorhinolaryngology, Head and Neck Surgery, Chang Gung Memorial Hospital and Chang Gung University, Taoyuan, Taiwan, R.O.C.; 4 Department of Pathology, Chang Gung Memorial Hospital and Chang Gung University, Taoyuan, Taiwan, R.O.C.; 5 Department of Radiation Oncology, Chang Gung Memorial Hospital and Chang Gung University, Taoyuan, Taiwan, R.O.C.; 6 Department of Medical Oncology, Chang Gung Memorial Hospital and Chang Gung University, Taoyuan, Taiwan, R.O.C.; 7 Department of Diagnostic Radiology, Chang Gung Memorial Hospital and Chang Gung University, Taoyuan, Taiwan, R.O.C.; 8 Nuclear Medicine and Molecular Imaging Center, Chang Gung Memorial Hospital and Chang Gung University, Taoyuan, Taiwan, R.O.C.; Georgia Regents University, College of Dental Medicine, United States of America

## Abstract

**Background:**

The fibula osteoseptocutaneous free flap is generally used for segmental mandibular reconstructions following resection of oral cavity squamous cell carcinoma (OSCC). However, less complex reconstructions may be feasible for patients with predicted poor survival. Herein, we sought to identify the main risk factors (RFs) associated with poor prognosis in OSCC patients undergoing segmental mandibulectomy to help decide between fibular and non-fibular reconstructions.

**Methods:**

Between 1996 and 2011, we examined the 5-year control, distant metastases, and survival rates in 310 consecutive, previously untreated patients with primary OSCC who underwent segmental mandibulectomy.

**Results:**

Margin status was the only independent RF for 5-year local control. Level IV/V metastases, extracapsular spread, and tumor depth ≥15 mm were independent RFs for poor 5-year survival. In the entire study cohort, 23% of the patients had 2 or 3 adverse RFs; such a high-risk group was characterized by a poor prognosis and may be suitable for non-fibular reconstructions. Overall, 70% of the study patients were cT1-4N0, cT1N2, cT2N1, or had tumor depth <15 mm; less than 5% of patients in this subgroup had 2 or 3 adverse RFs and were thus candidates for fibular reconstructions. Among the remaining 30% of patients who showed both advanced clinical stage (cT2N2, cT3-4N1-2) and tumor depth ≥15 mm, 70% exhibited 2 or 3 adverse RFs.

**Conclusions:**

Level IV/V metastases, extracapsular spread, and tumor depth ≥15 mm were independent predictors of poor prognosis in OSCC patients undergoing segmental mandibulectomy. The preoperative or intraoperative identification of adverse RFs may help decide between fibular and non-fibular mandibular reconstruction. High-risk patients bearing 2 or 3 adverse RFs have poor prognosis and should not be considered as candidates for fibular reconstructions.

## Introduction

Oral cavity squamous cell carcinoma (OSCC) is common in betel quid chewing areas like Taiwan, and 50% of such tumors occur at the buccal-alveolar ridge-retromolar trigone site [1]. Betel quid-associated submucous fibrosis with trismus is frequently observed in our OSCC patients; consequently, the involved buccal mucosa often adheres to the alveolar ridge and the tumor bridges the buccal-gum complex. The management of OSCC is largely surgical, and bony excision by mandibulectomy is frequently required when the tumor involves or approaches the alveolar ridge.

Marginal mandibulectomy is indicated when the tumor approaches or involves in the alveolar ridge but has not reached the marrow. Conversely, segmental mandibulectomy is feasible when the neoplasm involves the mandibular marrow, the bone of the edentulous mandible, the bone of the irradiated mandible, or in presence of severe mandibular adherences caused by the tumor. In general, the resectional defect can be addressed with one of the following two approaches: 1) a simple method where a reconstruction plate is used to bridge the mandibular defect and then covered with a soft-tissue-only flap; or, 2) a comprehensive but more complex method where a vascularized osteocutaneous flap is used to restore mandibular bone continuity and adjacent soft tissues losses (intraoral and/or facial). Less commonly, in presence of complex or composite defects, two-flap reconstructions may be required to achieve an adequate repair of both the bone and soft tissues. The fibula osteoseptocutaneous free flap is generally used for segmental mandibular reconstructions following OSCC resection. However, soft-tissue-only flap reconstructions (e.g., anterolateral thigh, vastus lateralis myocutaneous or radial forearm flaps) are less demanding and time-consuming than fibula osteoseptocutaneous free flap reconstructions. In this context, the former may be suitable for high-risk patients who have an adverse prognosis, whereas the latter can be recommended for patients with good predicted outcomes [2], [3]. Unfortunately, prognostic stratification still largely relies on subjective surgical judgments based on preoperative clinical and image findings. Patients requiring segmental mandibulectomy are generally considered at high risk because of the presence of “advanced” tumors (e.g., large tumors) and/or “advanced” nodal status (e.g., imaging findings indicating the presence of cN2 or extracapsular spread [ECS]). Notably, the impact of such risk factors on the clinical outcomes in the specific subset of OSCC patients requiring segmental mandibulectomy remains unclear.

In the present study, we sought to identify the main risk factors (RFs) associated with poor prognosis in OSCC patients undergoing segmental mandibulectomy to help decide between fibular and non-fibular reconstructions in a more evidence-based manner.

## Patients and Methods

This study was designed as a retrospective analysis of prospectively collected data. Since this study involved retrospective review of existing data, approval from the Institutional Review Board of the Chang Gung Memorial Hospital (CGMH) at Linkou (Number: 99-3131B, 101-4457B, and 102-2366C) was obtained, but without specific informed consent from patients. The study protocol was approved by the local Medical Ethics Committee with compliance to the guidelines of the Declaration of Helsinki. The written informed consent regarding detail information publication (as outlined in PLOS consent form) was also obtained from individual in this manuscript. All of the data were securely protected (by delinking identifying information from the main data sets), made available only to investigators, and analyzed anonymously.

This study was supported by grants No. CMRPG1B0591, Chang Gung Memorial Hospital. The funders had no role in the study design, data collection and analysis, decision to publish or preparation of the manuscript.

### Study Participants

Between January 1996 and July 2011, we prospectively enrolled 1570 consecutive, previously-untreated, first-primary OSCC patients who underwent radical tumor excision. Patients were collected in the clinicopathological database of the Head and Neck Oncology Team at Chang Gung Memorial Hospital in Linkou, Taiwan. For the purposes of this study, we retrieved from the database all of the patients who underwent segmental mandibulectomy as part of their OSCC resection (n = 310), either with or without neck dissection. Patients with suspected presurgical distant metastases were excluded. Patients were staged according to the 1997 (5^th^) and 2010 (7^th^) American Joint Committee on Cancer (AJCC) staging criteria [4], [5]. The 1997 staging system was used for patients enrolled before 2002, whereas the 2010 staging criteria were used thereafter. The main reason for the use of the AJCC 1997 criteria for patients presenting before 2002 instead of the AJCC 2010 staging system was that histopathological specimens collected before 2002 were not all available for additional review. Second, the correct staging of pT4b disease according to the AJCC 2002/2010 criteria might be troublesome for OSCC [6], [7]. The presurgical evaluation, primary definitive treatment and adjuvant therapy were performed as previously described [8].

### Data Analysis

Follow-up visits were continued until July 2013. All patients attended follow-up examinations for at least 24 months after primary surgery or until death. The study endpoints included the 5-year rates of local control, neck control, distant metastases, disease-free survival (DFS), disease-specific survival (DSS), and overall survival (OS). Local recurrence was defined as a tumor recurrence occurring at the primary tumor resection site. DFS was defined as the time from the date of surgery to the date of local, regional, distant progression, or the date of last follow-up. DSS was defined as the time from the date of surgery until death from OSCC or date of last follow-up. OS was calculated from the date of surgery to the date of last follow-up or death. The 5-year outcomes were computed using the product limit method of Kaplan-Meier and assessed for statistical significant with the log-rank test. Univariate and multivariate analyses were used to identify significant RFs. Independent prognostic factors were identified by a multivariate Cox regression analysis using the forward selection procedure. Statistical calculations were performed with SPSS software (version 17.0; SPSS Inc., Chicago, IL, USA). Two-tailed *P* values <0.05 were considered statistically significant.

## Results

### Patients

Table 1 summarizes the general characteristics of the 310 OSCC patients requiring segmental mandibulectomy. Of them, 125 underwent non-fibular reconstructions (non-fibular group), whereas 185 received fibular reconstructions (fibular group). The non-fibular group included 122 patients who received single-flap reconstructions (vastus lateralis myocutaneous flap, n = 118; pectoralis major myocutaneous pedicled flap, n = 3; forearm flap, n = 1) and 3 patients who had double-flap reconstructions (pectoralis major myocutaneous pedicled flap plus deltopectoral flap, n = 2; vastus lateralis myocutaneous flap plus forearm flap, n = 1), whereas the fibular group included 126 patients who had single-flap reconstructions and 59 who had double-flap reconstructions (fibular flap plus vastus lateralis myocutaneous flap, n = 55; fibular plus pectoralis major myocutaneous pedicled flap, n = 4). Compared with the fibular group, the non-fibular group included a higher number of patients with buccal subsite involvement, cT3-4 disease, cN2 disease, inferior maxillectomy, poor tumor differentiation, pT3-4 disease, pN2 disease, p-stage IV, ECS, level IV/V metastases, tumor depth ≥15 mm, skin invasion, adjuvant therapy, and a lower number of double free-flap reconstructions.

**Table 1:** The clinicopathological characteristics of OSCC patients treated by segmental mandibulectomy.

Characteristics (n, %)	Reconstruction		*P*
	Non-Fibular (n = 125)	Fibular (n = 185)	
	(n, %)	(n, %)	
Sex			0.518
Male (291, 93.9)	116 (92.8)	175 (94.6)	
Female (19, 6.1)	9 (7.2)	10 (5.4)	
Age (years), 25–85 (median 52)			0.191
<65 (263, 84.8)	102 (81.6)	161 (87.0)	
≥65 (47, 15.2)	23 (18.4)	24 (13.0)	
Tumor subsites			<0.001
Tongue (13, 4.2)	9 (7.2)	4 (2.2)	
Mouth floor (23, 7.4)	7 (5.6)	16 (8.6)	
Lip (3, 1.0)	2 (1.6)	1 (0.5)	
Buccal (109, 35.2)	59 (47.2)	50 (27.0)	
Alveolar ridge (133, 42.9)	36 (28.8)	97 (52.4)	
Retromolar (29, 9.4)	12 (9.6)	17 (9.2)	
Clinical T-status			0.003
cT1-2 (48, 15.5)	10 (8.0)	38 (20.5)	
cT3-4 (262, 84.5)	115 (92.0)	147 (79.5)	
Clinical N-status			0.001
cN0-1 (204, 65.8)	69 (55.2)	135 (73.0)	
cN2 (106, 34.2)	56 (44.8)	50 (27.0)	
Clinical Stage			0.148
I–III (62, 20.0)	20 (16.0)	42 (22.7)	
IV (248, 80.0)	105 (84.0)	143 (77.3)	
Two flaps reconstruction			<0.001
No (249, 81.6)	119 (99.2)	130 (70.3)	
Yes (56, 18.4)	1 (0.8)	55 (29.7)	
Free-flap reconstruction			<0.001
Single (246, 82.3)	117 (99.2)	129 (71.3)	
Double (53, 17.7)	1 (0.8)	52 (28.7)	
Inferior maxillectomy			0.004
No (217, 70.0)	76 (60.8)	141 (76.2)	
Yes (93, 30.0)	49 (39.2)	44 (23.8)	
Tumor differentiation			0.012
Well/moderate (279, 90.0)	106 (84.8)	173 (93.5)	
Poor (31, 10.0)	19 (15.2)	12 (6.5)	
Pathological T-status			<0.001
pT1-2 (58, 18.7)	11 (8.8)	47 (25.4)	
pT3-4 (252, 81.3)	114 (91.2)	138 (74.6)	
Pathological N-status^a^			0.002
pN0-1 (199, 64.4)	68 (54.4)	131 (71.2)	
pN2 (110, 35.6)	57 (45.6)	53 (28.8)	
Pathological stage^b^			0.007
I–III (63, 20.3)	16 (12.8)	47 (25.4)	
IV (247, 79.7)	109 (87.2)	138 (74.6)	
Extracapsular spread^a^			0.001
No (197, 63.5)	65 (52.0)	132 (71.4)	
Yes (113, 36.5)	60 (48.0)	53 (28.6)	
Level IV/V metastases			0.030
No (297, 95.8)	116 (92.8)	181 (97.8)	
Yes (13, 4.2)	9 (7.2)	4 (2.2)	
Tumor depth (mm) c ^,^ d			<0.001
<15 (153, 49.4)	46 (36.8)	107 (57.8)	
≥15 (157, 50.6)	79 (63.2)	78 (42.2)	
Margin status (mm) a			0.143
≤4 (33, 10.7)	17 (13.9)	16 (8.6)	
>4 (274, 89.3)	105 (86.1)	169 (91.4)	
Bone marrow invasion			0.448
No (147, 47.4)	56 (44.8)	91 (49.2)	
Yes (163, 52.6)	69 (55.2)	94 (50.8)	
Skin invasion			<0.001
No (245, 79.0)	86 (68.8)	159 (85.9)	
Yes (65, 21.0)	39 (31.2)	26 (14.1)	
Perineural invasion^a^			0.055
No (200, 64.7)	73 (58.4)	127 (69.0)	
Yes (109, 35.3)	52 (41.6)	57 (31.0)	
Vascular invasion^a^			0.532
No (299, 96.8)	120 (96.0)	179 (97.3)	
Yes (10, 3.2)	5 (4.0)	5 (2.7)	
Lymphatic invasion^a^			0.771
No (280, 90.6)	114 (91.2)	166 (90.2)	
Yes (29, 9.4)	11 (8.8)	18 (9.8)	
Treatment mode			0.033
S alone (74, 23.9)	22 (17.6)	52 (28.1)	
S plus RT/CCRT (236, 76.1)	103 (82.4)	133 (71.9)	

S, surgery; RT, radiotherapy; CCRT, concurrent chemoradiotherapy.

a Unavailable data: pN status (n = 1), margin (n = 3), perineural invasion (n = 1), vascular invasion (n = 1), lymphatic invasion (n = 1).

b Patient who did not receive neck dissection (n = 1) was classified as pN0.

c Optimal cut-off value for disease-free survival.

d Tumor depth was defined as the measured thickness from the surface of the normal mucosa to the deepest portion of the tumor.

### Five-year Outcomes after Segmental Mandibulectomy

The 5-year outcomes in the entire study cohort were as follows: local control, 81%; neck control, 88%; distant metastases, 18%; DFS, 64%; DSS, 71%; and OS, 51%. The 5-year second primary tumor rate was 26%. The 5-year outcomes of patients with non-fibular *versus* fibular reconstructions were as follows: local control, 75% *vs.* 85%, *P* = 0.0407; neck control, 82% *vs.* 91%, *P* = 0.0365; distant metastases, 25% *vs.* 13%, *P* = 0.0060; DFS, 55% *vs.* 70%, *P* = 0.0066; DSS, 57% *vs.* 80%, *P*<0.0001; and OS, 37% *vs.* 60%, *P*<0.0001, respectively.

### Clinical Course

The entire study cohort (n = 310) was followed up for a median of 43 months (mean: 57 months, range: 1–198 months). At the date of analysis, 134 (43%) were alive and 176 (57%) were dead. Only one patient undergoing fibular reconstruction died perioperatively (within 30 days of the operation). The patterns of recurrence were as follows: local (n = 50, 16%), neck (n = 33, 11%), and distant metastases (n = 50, 16%). Salvage therapy was performed in 27 (38%) of the 72 patients with local and/or neck recurrences. Among the patients who underwent salvage therapy, 15 (56%) were still alive when the data were analyzed, whereas the remaining 12 (44%) had died.

### Five-year Prognostic Factors

The following potential prognostic factors collected at the time of initial tumor presentation were examined to assess their prognostic significance for 5-year outcomes: sex, age of disease onset, cT-status, cN-status, clinical stage, fibular repair (yes *vs.* no), tumor differentiation, pT-status, pN-status, pathologic stage, ECS, level IV/V metastases, tumor depth, margin status, bone marrow invasion, skin invasion, perineural invasion, vascular invasion, lymphatic invasion, and treatment mode. Table 2 depicts the results of multivariate analyses for 5-year outcomes. Margin status was the only RF associated with 5-year local control. Level IV/V metastases, ECS, and tumor depth ≥15 mm were independent RFs for 5-year DFS, DSS, and OS rates.

**Table 2:** Multivariate analysis of 5-year outcomes in patients treated by segmental mandibulectomy (n = 310).

Risk factor^a^/ (n)	Local control	Neck control	Distant metastasis	Disease-free survival	Disease-specificsurvival	Overallsurvival
	*P*, HR (95%CI)	*P*, HR (95%CI)	*P*, HR (95%CI)	*P*, HR (95%CI)	*P*, HR (95%CI)	*P*, HR (95%CI)
Margin status ≤4 mm	0.001	ns	ns	0.002	ns	ns
(n = 33)	3.148			2.251		
	(1.559–6.356)			(1.331–3.809)		
pN2	ns	<0.001	ns	ns	ns	ns
(n = 110)		4.210				
		(1.915–9.253)				
Level IV/V metastases	ns	0.004	0.049	0.001	0.001	0.018
(n = 13)		4.499	2.417	3.165	3.222	2.225
		(1.629–12.422)	(1.005–5.813)	(1.556–6.436)	(1.567–6.625)	(1.148–4.312)
Extracapsular spread	ns	ns	<0.001	<0.001	<0.001	<0.001
(n = 113)			12.196	2.877	4.158	2.875
			(5.351–27.797)	(1.920–4.309)	(2.543–6.799)	(2.109–3.920)
Vascular invasion	ns	0.005	ns	ns	ns	ns
(n = 10)		5.627				
		(1.670–18.961)				
Poor differentiation	ns	ns	0.007	ns	0.040	ns
(n = 31)			2.412		1.830	
			(1.279–4.549)		(1.028–3.259)	
Tumor depth ≥15 mm	ns	ns	ns	0.042	0.004	0.016
(n = 157)				1.528	2.009	1.462
				(1.016–2.299)	(1.255–3.215)	(1.074–1.991)
Non-fibular reconstruction	ns	ns-	ns	ns	ns	0.004
(n = 125)						1.584
						(1.162–2.157)

HR: hazard ratio; CI: confidence interval; ns: not significant.

a All of the factors identified in univariate analysis were entered into the multivariate analyses; only significant factors were listed in this table.

### Scoring System for 5-year Outcomes

**Figure 1:**
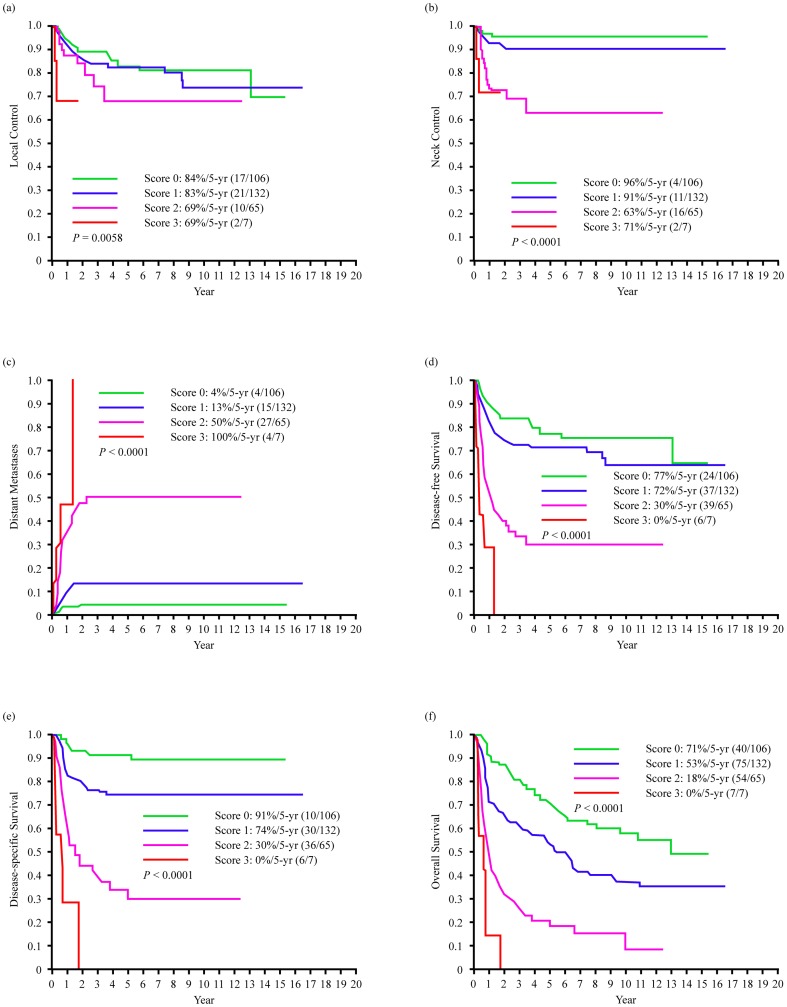
Kaplan-Meier estimates of 5-year outcome in the segmental mandibulectomy patients stratified by level IV/V metastases, extracapsular spread status and tumor depth of ≥15 mm, (a) local control, (b) neck control, (c) distant metastases, (d) disease-free survival, (e) disease-specific survival, (f) overall survival.

We then divided the study participants according to a prognostic scoring system formulated by summing up the three significant covariates identified as independent RFs in multivariate analysis (level IV/V metastases, ECS, and tumor depth ≥15 mm). Each of these factors was given a score of 1, resulting in a score of 0 in the absence of RFs, a score of 1 in the presence of one RF, a score of 2 in the presence of two RFs, and a score of 3 in the presence of three RFs. Figure 1 shows the 5-year control, distant metastases, and survival rates according to the prognostic scoring system (Fig. 1, a–f). Patients with scores of 0 (n = 106) or 1 (n = 132) had better 5-year survival rates than patients with scores of 2 (n = 65) or 3 (n = 7) (*P*<0.0001) (Fig. 1, d–f). Of note, patients with scores of 2 or 3 had a 5-year distant metastatic rate greater than 50% (Fig. 1, c).

### Clinical Staging versus Prognostic Scoring System

Table 3 (left) depicts the relationship between clinical staging and the proposed scoring system. Patients with cT1-4N0, cT1N2, or cT2N1 had a mean 3% (4/137) probability of having a score of 2 or 3, whereas other advanced stage patients (cT2N2, cT3-4N1-2) showed an approximately 40% probability of (68/173) of scoring 2 or 3. Table 3 (right) shows the relation between clinical staging and the proposed scoring system stratified according to the presence of a tumor depth ≥15 mm on both sides. Patients with a tumor depth <15 mm had a mean 3% (4/153) probability of scoring 2 or 3, whereas those with tumor depth ≥15 mm and cT2N2, cT3-4N1-2 had approximately a 70% (64/96) chance.

**Table 3:** Scoring system of OSCC patients in different clinical stages with and without stratification for tumor depth (cut-off: 15 mm).

Clinicalstaging	Scoring				Stratification according to tumor depth			
					depth <15 mm		depth ≥15 mm	
(n)	score 0	score 1	score 2	score 3	score 0–1	score 2	score 1	score 2–3
	(n, %)	(n, %)	(n, %)	(n, %)	(n)	(n)	(n)	(n)
T1N0 (1)	1 (100.0)	-	-	-	1	-	-	-
T2N0 (23)	19 (82.6)	4 (17.4)	-	-	20	-	3	-
T3N0 (19)	9 (47.4)	9 (47.4)	1 (5.3)	-	9	-	9	1
T4N0 (83)	33 (39.8)	48 (57.8)	2 (2.4)	-	36	-	45	2
T1N2 (1)	-	1 (100.0)	-	-	1	-	-	-
T2N1 (10)	5 (50.0)	4 (40.0)	1 (10.0)	-	9	-	-	1
T2N2 (12)	4 (33.3)	4 (33.3)	4 (33.3)	-	8	2	-	2
T3N1 (9)	2 (22.2)	2 (22.2)	5 (55.6)	-	3	-	1	5
T3N2 (5)	-	2 (40.0)	2 (40.0)	1 (20.0)	1	-	1	3
T4N1 (59)	21 (35.6)	27 (45.8)	11 (18.6)	-	31	-	17	11
T4N2 (88)	12 (13.6)	31 (35.2)	39 (44.3)	6 (6.8)	30	2	13	43
Total (310)	106 (34.2)	132 (42.5)	65 (21.0)	7 (2.3)	149	4	89	68

## Discussion

Mandibulectomy is frequently required for OSCC tumor ablation. A review of the literature revealed that only a few studies have been conducted to analyze patient outcomes following mandibulectomy [9]–[12]. Notably, their main focus was on the differences in local control and survival outcomes between marginal versus segmental mandibulectomy. There have been no previous studies investigating the outcomes of patients who received different reconstruction methods according to a preoperative evaluation of disease severity. Compared with other osseous flaps (e.g., iliac crest and scapular flaps), the free fibula osteoseptocutaneous flap is generally considered as the optimal option for the reconstruction of segmental bony mandibular defects. However, fibular reconstructions are technically demanding and time-consuming and may be thus unfeasible for high-risk subjects with a predicted negative outcome. In this study, we examined disease control and survival rates in patients who underwent segmental mandibulectomy with the aim of identifying the main prognostic RFs that may be used for prioritizing osteoseptocutaneous free flap reconstructions.

In general, the decision to perform fibular vs. non-fibular reconstructions is primarily based on the surgeon's clinical assessment, patient history, physical examination, and results of preoperative investigations. Of note, the choice of reconstruction may be changed intraoperatively if the actual disease severity differs from that predicted severity based on preoperative findings. In this study, we found that the non-fibular group included a higher number of patients with cT3-4 and cN2 disease (Table 1), whereas a greater number of subjects who underwent two-flap reconstructions was noted in the fibular group. The latter also had more patients with tumors at the alveolar ridge, whereas the former showed a greater prevalence of tumors located at the buccal subsite. The postoperative findings confirmed the preoperative data showing that the non-fibular group included more patients with poor tumor differentiation, pT3-4 status, pN2 status, p-stage IV disease, tumor depth ≥15 mm, and skin invasion. The presence of such RFs was paralleled by clinical outcomes. Accordingly, the 5-year outcomes of patients who underwent non-fibular reconstructions were significantly worse than those of subjects who received fibular reconstructions. These results confirmed the clinical usefulness of the surgeon's subjective assessment on the use of fibular vs. non-fibular reconstructions after segmental mandibulectomy.

The results of multivariate analyses indicated that the margin status was the only RF associated with the 5-year local control rate. Level IV/V metastases, ECS, and tumor depth ≥15 mm were independently associated with 5-year DFS, DSS, and OS. Notably, neither c-TNM (cT-status, cN-status, clinical stage) nor p-TNM staging (pT-status, pN-status, pathologic stage) were independently associated with survival rates (Table 2). Patients with less severe cN+ (Table 3 [left]: T1-4N0, T1N2, T2N1, 3% probability of a score 2−3) or a lower tumor depth (Table 3 [right]: <15 mm, 3% probability of a score 2−3) may be candidates for fibular reconstruction due to their expected positive outcomes. These two patient groups comprised about 70% (214/310) of the entire study cohort. Approximately 70% (64/96) of patients with advanced clinical tumor stages (cT2N2, cT3-4N1-2) and a tumor depth ≥15 mm had poor prognostic scores (2−3); this subgroup may require additional evaluations for establishing the priority of fibular vs. non-fibular reconstructions. Although cN2 was significantly associated with 5-year DFS, DSS, and OS rates in univariate analysis (all *P*<0.0001, data not shown), this RF did not retain its independent prognostic significance in multivariate analysis.

**Figure 2:**
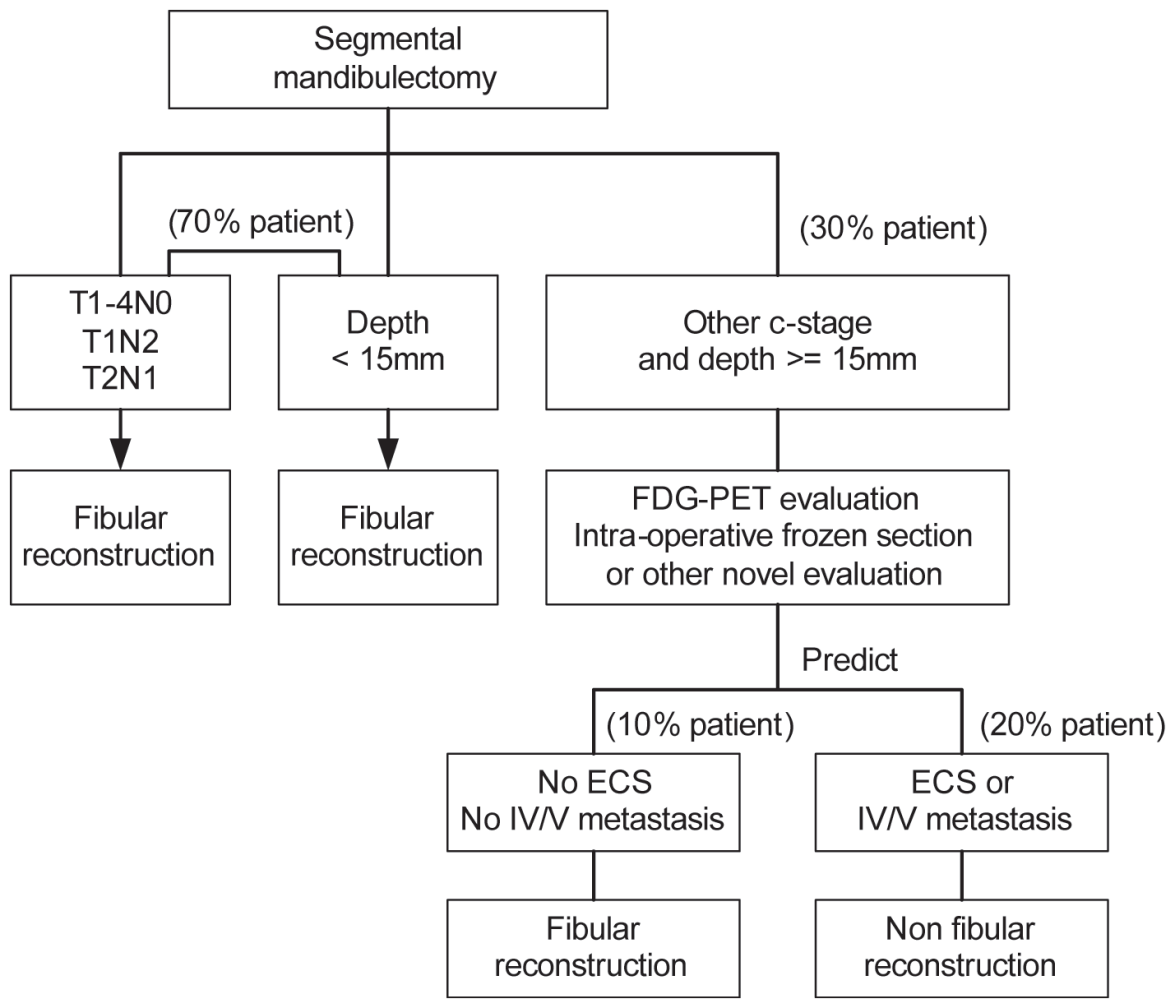
Treatment decision tree for fibular reconstruction.

As of 2002, most of our patients underwent preoperative FDG-PET imaging. Two of our previous FDG-PET reports have suggested the potential occurrence of ECS or level IV/V metastases [13], [14]. In one study, we have shown that a preoperative FDG-PET standardized uptake value of the neck lymph nodes (SUVnodal) ≥5.7 identified approximately 90% of patients with pathological ECS [13]. In this study, 49 patients had a SUVnodal value of ≥5.7, and 86% (44/49) of them had pathological ECS. In the second report, we demonstrated that FDG-PET of the neck lymph nodes improves risk prediction in OSCC patients [14]. The results from this study also demonstrated that 60 of the 72 patients (83%) who had a SUVnodal visual score >4 had proven pN+ disease. Intraoperative frozen section analyses of suspicious lymph nodes may confirm the presence or absence of ECS or level IV/V metastases and thus influence the decision of performing gold-standard fibular reconstruction. The presence of a tumor depth ≥15 mm may be suggested or diagnosed by preoperative imaging, despite the possibility of a mean 30% tissue shrinkage [15]. Based on our findings, we propose a scoring system for devising the optimal reconstruction method for OSCC patients undergoing segmental mandibulectomy (Figure 2). The importance of close or positive margins, also identified by other groups [9]–[12], confirms that great efforts must be taken to obtain clear margins when performing segmental mandibulectomy.

When we divided OSCC patients based on the presence of RFs (level IV/V metastases, ECS, and tumor depth ≥15 mm), patients with scores of 0 or 1 had better 5-year survival rates than those with a score of 2 or 3 (Fig. 1, d–f). Patients who scored 2 or 3 had a 5-year rate of distant metastases greater than 50% (Fig. 1-c). These results suggest that patients with scores of 2 or 3 are not suitable candidates for fibular flap reconstruction. However, there may be ethical concerns in limiting highly functional reconstructions only to patients with predicted good outcomes. A potential solution would be to delay a second fibular flap reconstruction at least 2 years after the initial period characterized by a high risk of distant metastases. In general, fibular reconstructions should be recommended for OSCC patients undergoing segmental mandibulectomy with a preoperative risk score of 0 or 1. In contrast, non-fibular reconstructions should be proposed to patients with a score of 2 or 3 before or during radical surgery with curative intent.

In the current study, we determined the indications for fibular reconstruction after segmental mandibulectomy based primarily on preoperative objective disease severity and outcome data. An important limitation inherent in our study is that all of the patients had undergone reconstructive surgery, making difficult to weight the relative contribution of RFs versus that of reconstructive surgery per se. Therefore, the clinical utility of our RFs needs independent validation in a well-designed prospective study. Moreover, the lack of data on the patient's general medical and anaesthetic conditions (that may affect the choice of reconstruction independent of OSCC-related control or survival outcomes) is another caveat of our report. Future studies focusing on mandibular reconstruction may also include intraoperative frozen section analyses of nodal tissues. Importantly, great efforts should be generally required to obtain clear margins.
